# Conceptions of food healthiness among nutrition and food science undergraduates: A mixed-methods study in a Spanish university

**DOI:** 10.1371/journal.pone.0344433

**Published:** 2026-03-10

**Authors:** Ricard Celorio-Sardà, Mari Aguilera, Claudia Soar, Maria Clara Gómez, Oriol Comas-Basté, M. Carmen Vidal-Carou, Maria Clara de Moraes Prata Gaspar

**Affiliations:** 1 Departament de Nutrició, Ciències de l'Alimentació i Gastronomia, Campus de l'Alimentació de Torribera, Universitat de Barcelona (UB), Santa Coloma de Gramenet, Spain; 2 Institut de Recerca en Nutrició i Seguretat Alimentària (INSA·U.B.), Universitat de Barcelona (UB), Santa Coloma de Gramenet, Spain; 3 Departament de Cognició, Desenvolupament i Psicologia de l'Educació, Secció Cognició, Facultat de Psicologia, Universitat de Barcelona (UB), Barcelona, Spain; 4 Institut de Neurociències (UBNeuro), Universitat de Barcelona (UB), Passeig de la Vall d'Hebron, Barcelona, Spain; 5 Nutrition Post-Graduate Program, Department of Nutrition, Federal University of Santa Catarina, Florianópolis, South Carolina, Brazil; 6 Departament d'Antropologia Social, Facultat de Geografia i Història, Universitat de Barcelona (UB), Barcelona, Spain; Lusofona University of Humanities and Technologies: Universidade Lusofona de Humanidades e Tecnologias, PORTUGAL

## Abstract

Understanding how future nutrition and food professionals conceptualize healthy food and eating is key to aligning university training and professional practice with public health and sustainability goals. This mixed-methods study explored how undergraduate students of Human Nutrition and Dietetics and of Food Science and Technology at a Spanish university define what makes foods healthy, and how these views differ by degree, gender, and year of study. The qualitative phase was based on two focus groups (n = 13) while the quantitative phase used a structured online questionnaire distributed across all academic years (n = 300). Students described healthy eating through moderation, variety, and nutrient balance, consistently elevating fruits, vegetables, legumes, nuts, and olive oil while positioning sugary drinks, sweets, and highly processed products as less healthy. Disciplinary contrasts emerged for animal-derived foods: Nutrition students judged red and processed meats more negatively than their peers in Food Science. With academic progression, perceptions tended to show a more favorable views of fish, olive oil, nuts, and fermented foods. A reduced reliance on claims such as “organic” or “GMO-free” was also observed. Women placed greater emphasis on plant-based choices, wholegrains, seasonality, proximity, and animal welfare, whereas men evaluated meats and alcoholic beverages more positively and expressed stronger trust in official quality seals. Agreement was highest with biomedical and holistic meanings of food, while endorsement of sociocultural definitions declined over the course of study. These findings provide insights into the evolving professional identities of future nutritionists and food technologists and support the importance to integrate more deeply sociocultural and sustainability perspectives into university curricula.

## Introduction

The concept of “healthy food” permeates medical, political, media, and lay discourses, reflecting its multifaceted nature in contemporary societies [1–3]. Although there is broad scientific consensus regarding the health-promoting or detrimental characteristics of certain foods [4], food-health associations are not solely grounded in objective nutritional knowledge [5–7]. Rather, these associations are shaped by particular contexts, involving interactions among social values, representations, practices, and norms that organize social life [8,9].

Food categorization systems vary historically and socioculturally, with each culture defining its criteria for classifying foods as edible, indigestible, good, toxic, or healthy [10,11]. These categorizations are internalized by individuals within their respective social groups, significantly influencing their social identity construction [12]. Thus, the notion of “healthy food” emerges as a sociocultural and scientific construct, blending lay and expert knowledge, and transforming across historical and spatial contexts, including contemporary Western societies [6,11].

Scrinis (2013) describes nutritionism as a reductionist paradigm that emerged in the 1960s, framing foods primarily through their nutritional and health dimensions [13]. One of its key features is the binary categorization of nutrients and foods as “good” or “bad”. This construction often results in current moral judgements, where “good” foods symbolize nourishment, self-control, and health-consciousness, whereas “bad” foods represent health risks and moral weakness [11]. Such distinctions are deeply embedded within cultural values and social group expectations, reinforcing moral dimensions in dietary choices [14].

Although expert discourse has since evolved toward more holistic approaches emphasizing dietary patterns rather than individual foods, such dichotomies continue to influence both public and professional understandings of healthy eating [6,13]. Previous studies examining perceptions of food healthiness have shown that these simplified categorizations remain deeply embedded in how different populations interpret and evaluate foods [14,15]. Consumer perceptions of healthiness are influenced by sensory and symbolic cues, including those embedded in branding, language, and product presentation. Research in sensory marketing has shown that product-extrinsic factors, such as brand names, packaging design, and even the phonetic qualities of words can subtly yet significantly affect perceived healthfulness [11]. Social contexts further amplify these effects: norms transmitted through peers, family, and broader cultural networks can reinforce or challenge individual dietary beliefs [12].

Current research also emphasizes the increasing importance of sustainability considerations in definitions of healthy food and eating [7,16,17]. Sustainable diets are characterized by low environmental impact, accessibility, and cultural acceptability, alongside nutritional adequacy [18,19]. Public health campaigns and educational efforts have begun integrating sustainability criteria into dietary guidelines, reflecting a shift towards holistic dietary recommendations that simultaneously address health, environmental, and social dimensions [20–22]. These shifts align with broader planetary health frameworks and the One Health perspective [17,23].

Few studies have explored how social representations concerning food and a healthy food are shaped within academic contexts, especially in Spain and particularly among future nutrition and food science professionals, an evidence gap that this study aims to address. Previous research indicates significant variability in perceptions among dietetics and food science students, influenced by their academic training and sociocultural contexts [6,24]. Furthermore, most studies examining perceptions of healthy eating have predominantly limited their approach to quantitative methodologies, typically employing closed questionnaires or focusing on food hierarchies [1,5,25].

University students, specifically those in food and nutrition sciences, represent a particularly relevant population, as their perceptions can influence future professional practices and public health initiatives [26]. Therefore, given the pivotal role that food and health professionals play in promoting dietary recommendations and influencing public perceptions, it is critical to understand how these groups conceptualize “healthy food.” Therefore, this study aims to explore and analyze social perceptions of food healthiness among undergraduate students enrolled in Human Nutrition and Dietetics (HND) and Food Science and Technology (FST) degrees at the University of Barcelona (UB) from Spain. Furthermore, this study examines how these perceptions vary according to disciplinary background, gender, and academic year. By combining qualitative focus group data with quantitative survey analyses, this mixed-methods approach allows for an integrated examination of both the meanings students attribute to “healthy food” and the patterns of evaluation they apply to different foods and attributes. In doing so, the study goes beyond previous research that has typically addressed these dimensions separately, providing a comprehensive perspective on the joint influence of professional socialisation, gendered norms, and academic progression on conceptions of food healthiness within a Southern European university context still little explored in the scientific literature.

## Materials and methods

### Study design

This paper presents a secondary study derived from data collected in an exploratory and descriptive cross-sectional study conducted during the first half of 2021 by an interdisciplinary research team [27]. It specifically analyzes student perceptions of food healthiness, applying both qualitative and quantitative methodologies, to provide comprehensive insights into this complex and context-dependent phenomenon.

A mixed-methods design is particularly appropriate for the study of social representations of food healthiness, as these are multidimensional and socially constructed constructs that encompass both shared meanings and measurable patterns of evaluation. In this sense, combining qualitative and quantitative methodologies allows for the collection of complementary types of data on the same object of study, which can be contrasted to produce a more comprehensive analysis that captures the complexity of the phenomenon under investigation [28]. The qualitative phase enabled an in-depth exploration of students’ discourses, categories, and underlying rationalities, which informed both the development and interpretation of the quantitative instrument. In turn, the survey allowed for the systematic comparison of these representations across degree programs, genders, and academic years, thereby integrating depth of understanding with analytical generalisability.

The study followed the ethical standards of the Declaration of Helsinki and received approval from the Bioethics Commission of the University of Barcelona (protocol IRBOC003099, 2020). Written informed consent was obtained from all individual participants included in the study, data confidentiality was guaranteed, and the names of the informants cited in this article are fictional. No minors participated in the current study.

### Setting and sample

The study was conducted using a convenience sample of male and female students, recruited between February 1 and June 15, 2021, enrolled in the four academic years of two bachelor's degrees: HND and FST, at the UB, a leading institution in nutrition and food science education in Spain. As defined by the university, the HND degree trains professionals to promote adequate nutrition tailored to physiological and pathological needs, including dietary and nutritional strategies for the treatment and prevention of disease. In contrast, the FST degree prepares students for technical roles related to food production, packaging, preservation, and innovation, in alignment with food safety, quality, and sustainability standards. There was no exclusion criteria based on age, nationality, or place of residence.

### Data production and analysis

The qualitative phase of this study was based on two focus groups (n = 13) and is reported following the Standards for Reporting Qualitative Research (SRQR). The aim of these focus groups was primarily to provide an exploratory entry into the field in order to gain an initial understanding of students’ conceptions for the subsequent design of the questionnaire, rather than to seek representativeness or generate a closed theoretical framework. Each session of the focus groups lasted approximately 90 minutes and was moderated by a researcher specialised in the socio-anthropology of food, with assistance from a dietitian postgraduate student in anthropology. The discussions were guided by an interview script designed for the study and included open-ended questions related to food perceptions, trust, food practices, sustainability, and diet. During the focus groups, the moderator actively encouraged balanced participation and minimized the influence of dominant speakers. Data saturation was assessed pragmatically, as no substantially new themes emerged across the two groups and the main discursive axes were reiterated. The sessions were recorded with participant consent and transcribed verbatim.

Thematic content analysis was conducted using Atlas.Ti (version 8, Visual Qualitative Data Analysis, 2017). Analytical categories were defined by the research team to systematise the collected data through both inductive and deductive processes, informed by a prior literature review, the study objectives and in‐ depth readings of all interview transcripts: perceptions of healthy food and eating, perceptions of food, trust/distrust of food, food sustainability, culinary activity, food choices, vegetarianism, and changes in perception during the degree studies. The qualitative findings were subsequently contrasted and expanded through the quantitative phase of the study.

The insights from this qualitative phase, together with data from previous studies [27,29], contributed to the construction of a 31-item questionnaire with multiple choice and Likert-scale questions. This questionnaire underwent expert review and validation by 20 professionals in nutrition (n = 12), sociology and anthropology (n = 7), and statistics (n = 1). These professionals were asked to evaluate the clarity, objectivity, and relevance of the content of each question and of the questionnaire as a whole. A pilot test was carried out with 30 students, who were invited to comment on the questionnaire after completing it. The comments from the pilot test participants were used to make the final adjustments to the questionnaire content.

The final version was administered online via SurveyMonkey between April and May 2021. A total of 385 responses were collected, of which 300 were complete and valid (78.0% completion rate). Among the respondents, 151 were students of HND and 149 of FST, representing 47.0% and 45.0% of total enrolment in those degrees of the university of Barcelona. The average age was 21.25 years (SD = 3.16), with higher female representation (80.3%). Additional sample details and the full questionnaire used in this study can be found in Gaspar et al. (2023) [29]. From the original questionnaire, the present article focuses on four different questions (Q18, Q29-Q31) that were designed to assess the perception of students regarding the concept of food and their conceptualization of what makes a food healthy.

Data analysis was conducted using JASP statistical software (version 0.19.3). Descriptive statistics were expressed as means (M) and standard deviations (SD). Group comparisons were performed using independent samples t-tests, applying Welch’s correction when the assumption of equal variances was violated, and one-way ANOVA when comparing more than two groups. When ANOVA results indicated statistically significant differences, Tukey’s HSD post-hoc tests were applied. Statistical significance was set at p < .05. Effect sizes were computed as Cohen’s d for t-tests and η² for ANOVA. Cohen’s d values of ~0.20, ~ 0.50 and ~0.80 are interpreted as small, medium and large effects, respectively. Values of η² of ~0.01, ~ 0.06 and ~0.14 are similarly considered small, medium and large. Both are always reported as absolute values.

## Results

### Perceptions of a healthy food: qualitative results

A central topic of the discussion groups was the definition of what constitutes a healthy food and how to establish this type of classification. According to analyses, when students talk about healthy food, they generally conclude that anything can be healthy depending on the dose, that is, the amount consumed and the frequency:

“It also depends on the dose, right? That you take of the food itself.” (Alba, FST)

“A healthy food… I don’t think I can actually define it. Everything can be healthy in the right amount…” (Amanda, HND)

Appropriate consumption of each food, without excesses or deficiencies, was said to ensure variety, a fundamental aspect of a healthy diet, along with correct and balanced nutrient intake. It would also help avoid excessive intake of certain substances added during production (e.g., sweeteners, colourings), which are perceived as harmless if consumed in moderation:

“Depending on how much colouring it contains, it won’t do anything to you, or depending on the amount, it might. So yes, it can be healthy, but if you go overboard, then it’s not. Still, it’s not harmful.” (Laura, FST)

“In the case of Coca-Cola, there are substances that may not be entirely beneficial to our bodies, but we have systems that excrete these less desirable compounds. The problem is when, like with sugar, you eat a lot of pastries or sweets very regularly, there comes a point when your body can’t handle it anymore. It can’t regulate them. But as Clara said about the food pyramid: if it’s at the top, it means it can be consumed. Nothing bad will happen if you consume it but it should be consumed only occasionally. So it’s healthy, but it’s healthy to eat it once a month, not regularly.” (Gemma, HND)

Students also considered whether a food is healthy depends on the individual, their needs and goals, the interaction between the food and the body, and how it is combined with other dietary components:

“A professor once told us, ‘In nutrition, everything is relative.’ Is this food healthy? Well, it’s relative. It depends on the quantity, age, what you mix it with...” (Elena, FST)

“Is it healthy or not? It depends, it’s important to consider the interaction between the food and the body. A food is just a food, and it’s only healthy when it interacts with the body.” (Eric, FST)

“Peanut butter in large amounts isn’t very healthy, but it depends on your characteristics and your goals, a bit on your needs too, and if you control the quantity, then yes, it’s good and has benefits.” (Carlos, HND)

Although students aimed to relativize the definition of healthy food, they often used words like health and nutritionally adequate, sidelining the pleasure derived from food and emphasising its biological function:

“For me, a healthy food is one that is nutritionally more complete than another.” (Anna, FST)

Even though some, especially first-year students, showed inconsistencies when defining healthy food, most of them referred to something that benefits health:

“A healthy food is one that will sit well with you and is nutritionally balanced, not just one nutrient or one vitamin…” (Marta, HND)

“A healthy food for me is one that contains a balance of nutrients that, generally speaking, could be biologically good for someone’s health.” (Clara, FST)

Moreover, while they emphasised the importance of frequency and portion size, their discourse revealed that some products were perceived as more or less healthy than others, which highlights contradictions in their perceptions:

“Some foods are clearly unhealthy. I’m thinking of cooking oil that’s been reused too much. That’s definitely not healthy for anyone, not even in small amounts.” (Eric, FST)

In general, fruits, vegetables, and wholegrain products were perceived as healthy, while foods high in fat and sugar, as well as processed products, were seen as less healthy:

“For me, a healthy food product is one that provides more benefits, like fruit, the usual stuff, vegetables… not, like, cereals from Mercadona that are processed with loads of sugar.” (Mireia, HND)

“I think subconsciously I do pay attention to the nutritional aspect... I really try to notice whether I’m eating wholegrain products or not… whether my eating habits align with what I study every day.” (Amanda, HND)

The origin of food ingredients also shaped perceptions of healthiness:

“You see sugar, but I see everything else, where it comes from, not just the food matrix. For me, sugar from a legume is much healthier than sugar from a can of Coca-Cola.” (Samanta, FST)

The production method and processing techniques were also highlighted as relevant factors:

“It depends on the quantity... Frequency, quantity, processing method... because the final product can be made in different ways... all of that matters.” (Anna, FST)

Industrial food products, often referred to by students as “processed” or “ultra-processed”, were frequently perceived as less healthy than more “natural” alternatives, largely due to added “chemical” substances, which generated mistrust. It is noteworthy that the term “ultra-processed foods” appeared in some participants’ narratives as part of their spontaneous descriptions of foods perceived as unhealthy. This term was not introduced by the researchers nor operationalised as a predefined classification category within the study, but is reported here as an element of students’ own language and perceptions. On the contrary, some of the students seem to place their trust in products perceived as coming from smaller, local producers and more artisanal processes, which result in a final product with fewer ingredients:

“In the food industry, there are no limits, even though it’s well known that combining refined flours, sugars, and trans fats is super harmful, for example, it raises insulin levels…” (Samanta, FST)

“Coca-Cola isn’t healthy because aside from the food itself, it also contains sweeteners, colourants, sugar, carbonation… It’s got way more stuff than just water, colour, and a bit of sugar, there’s a lot behind Coca-Cola, which is why it’s not healthy.” (Marta, HND)

“Right no,w I try to eat lots of natural, unprocessed products. That’s what I focus on, also avoiding too much fat, especially the bad kind. (...) Because processed foods have a lot of chemicals that aren’t good for my body.” (Mireia, HND)

In some cases, organic production methods were also considered more natural, healthy, and trustworthy, but not all participants agreed with this view:

“The more organic, the better, because the flavour also changes, it’s more natural.” (Marta, HND)

“I completely disagree with Samanta about organic products. Organic doesn’t necessarily mean healthier or safer. In fact, there have been issues with organic foods because they often reuse animal manure, which contaminates the fruit. With other fertilisers, it might have been less organic but safer.” (Eric, FST)

Among FST students, there was a greater level of trust toward industrial food processing methods, which may be a direct consequence of the education they receive and the professional role they will have in relation to the food industry:

“Substances like preservatives, colourants… in the amounts used, they’re harmless. Like, on a chewing gum packet, it says that if you eat too many, you could get diarrhoea from the… what’s it called… from the sweeteners. But the amounts in products are regulated, they won’t do anything, they’re just preservatives, antioxidants, that kind of thing.” (Anna, FST)

“Personally, yes. I do trust the industry. If I didn’t, I wouldn’t be studying this degree.” (Eric, FST)

Overall, perceptions of students of what a healthy food is combined multiple rationalities, integrating nutritional and physiological criteria with more subjective, symbolic, moral, and ethical considerations. Beyond nutritional composition, they valued factors such as origin, production methods, processing level, and trust in producers or brands, often associating natural and local products with greater healthiness.

### Quantitative results

#### Definition of food.

Before analysing students’ perceptions of what makes a food healthy, it was considered important to first explore how they define food itself and the reasoning that underpins these definitions. Understanding these initial conceptions allows for a more nuanced interpretation of their later evaluations of food healthiness, as it sheds light on the rationalities and reference frames guiding their perceptions and choices.

Students were asked to indicate, through a five-point Likert scale, their level of agreement with four definitions of the concept of “food,” each reflecting different dimensions: one rooted in physiological and nutritional functions, two aligned with symbolic and sociocultural interpretations, and one incorporating physical, mental, and emotional health.

**Fig 1:**
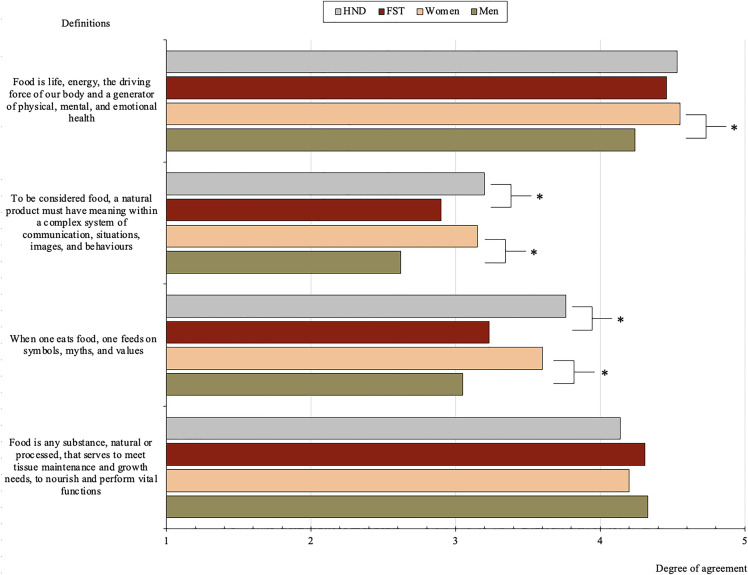
Degree of agreement with the different definitions of food by degree and gender. * p < .05.

Results show high levels of agreement with all four definitions, although variations in emphasis were identified. The highest average score among both groups was for the statement “Food is life, energy, the driving force of our body and a generator of physical, mental, and emotional health”, with mean scores of 4.53 (SD = 0.69) for HND students and 4.46 SD = 0.78) for FST students. There were no differences in the degree of agreement for this definition between both degrees (Fig 1).

Similarly, strong agreement was observed for the technically oriented definition: “Food is any substance, natural or processed, that serves to meet tissue maintenance and growth needs, to nourish and perform vital functions”. Mean values were 4.14 (SD = 1.06) for HND and 4.31 (SD = 0.96) for FST, with no statistically significant differences between degrees (Fig 1).

By contrast, definitions grounded in symbolic and cultural perspectives received comparatively lower agreement, especially among FST students. The statement “When one eats food, one feeds on symbols, myths, and values” scored 3.76 (SD = 1.05) among HND students and 3.23 (SD = 1.17) among FST students. Likewise, the definition “To be considered food, a natural product must have meaning within a complex system of communication, situations, images, and behaviours” yielded mean scores of 3.20 (SD = 1.16) for HND and 2.90 (SD = 1.16) for FST, indicating lower endorsement. Significant differences between the groups were found for these two culturally rooted definitions. HND students agreed more strongly with the idea that food involves a symbolic dimension (t = 4.144, p < .001, d = 0.48), a moderate effect size that suggests a meaningful difference in how students from the two degrees conceptualize food. By contrast, the smaller effect observed for the definition that a food product should carry communicative meaning to be considered food indicates a statistically reliable but more limited practical difference between degrees. (Fig 1).

Among HND students, statistically significant differences between years of the degree were observed only for the holistic health definition (F = 4.993, p = .008, η² = 0.06). First-year students (M = 4.67, SD = 0.67) and second/third-year students (M = 4.61, SD = 0.53) expressed greater agreement with this definition than fourth-year students (M = 4.26, SD = 0.85) (see Fig 1). The small-to-moderate effect size suggests that, although differences across academic years are not large, there is a consistent decline in endorsement of a holistic view of food as students progress through the degree.

In contrast, among FST students, statistically significant differences by year were found for three out of the four definitions. For one of the sociocultural definitions, there were significant differences (F = 9.028, p < .001, η² = 0.11), with first-year (M = 3.65, SD = 1.18) and second/third-year students (M = 3.27, SD = 1.00) showing greater agreement than fourth-year students (M = 2.66, SD = 1.18). The effect size for this definition (η² = .11) reflects a moderate effect of academic year, indicating a substantive reduction in the endorsement of sociocultural conceptions of food as students progress through the degree. For the other sociocultural definition, there was also a significant effect of the year (F = 5.051, p = .008, η² = 0.07). Agreement was higher among first-year students (M = 3.27, SD = 1.22) than among fourth-year students (M = 2.51, SD = 0.95) (Fig 1). This effect represents a small-to-moderate difference across academic years, again suggesting a gradual narrowing of symbolic interpretations of food in the later stages of the program.

For the holistic health definition, and as shown in Fig 1, differences were also significant (F = 3.332, p = .038, η² = 0.04), with a decline in agreement from first-year (M = 4.57, SD = 0.84) and second/third-year students (M = 4.54, SD = 0.65) to fourth-year students (M = 4.20, SD = 0.81). Although statistically significant, the small effect size suggests that differences across years are modest, indicating a subtle but consistent decline rather than a sharp conceptual shift.

The comparison of agreement levels between male and female students for the four definitions of food also revealed statistically significant gender differences in three out of the four statements. For the definition incorporating physical, mental, and emotional health, female students reported higher agreement (M = 4.55, SD = 0.69) compared to male students (M = 4.24, SD = 0.84), a statistically significant difference (t = 2.927, p = .004, d = 0.15) reflecting a small effect size and indicating a reliable but modest gender difference (Fig 1). This was also the highest-rated definition among women.

Similarly, women expressed significatively greater endorsement of the first symbolic and sociocultural definition (M = 3.60, SD = 1.13) compared to men (M = 3.05, SD = 1.10) (t = 3.357, p < .001, d = 0.49), a moderate effect size suggesting a meaningful gender difference in the symbolic framing of food. The same pattern emerged for the second symbolic and sociocultural definition, where women scored higher (M = 3.15, SD = 1.15) than men (M = 2.62, SD = 1.15) (t = 3.175, p = .002, d = 0.46), again reflecting a moderate effect. By contrast, no significant gender differences were observed for the physiological and nutritional definition (see Fig 1). However, this was the highest-rated definition among men (M = 4.33, SD = 0.94).

#### Perceived healthiness of foods.

Students were asked to rate the perceived healthiness of 19 different food groups on a 5-point Likert scale, where 1 indicated “not healthy at all” and 5 indicated “very healthy.” Overall, both HND and FST students showed very similar patterns in how they classified foods as healthy or unhealthy, with some statistically significant differences between groups (see Table 1).

**Table 1:** Perceived healthiness of food items by degree, year, and gender.

Food item	HND (n = 151)	FST (n = 149)		HND				FST				Female (n = 241)	Male (n = 58)	
	(M ± SD)	(M ± SD)	t	1^st^ (n = 46) (M ± SD)	2^nd^/3^rd^ (n = 61) (M ± SD)	4^th^ (n = 44) (M ± SD)	F	1^st^ (n = 49) (M ± SD)	2^nd^/3^rd^ (n = 59) (M ± SD)	4^th^ (n = 41) (M ± SD)	F	(M ± SD)	(M ± SD)	t
Red meat	2.16 ± 1.04	2.90 ± 1.05	−6.10***	2.85 ± 1.19	1.92 ± 0.71	1.77 ± 0.92	18.04***	3.94 ± 0.92	4.44 ± 0.79	4.24 ± 0.97	4.30*	2.40 ± 1.08	3.05 ± 1.08	−4.11***
White meat	3.59 ± 0.96	3.62 ± 0.98	−0.28	3.76 ± 1.16	3.38 ± 0.78	3.72 ± 0.93	2.67	4.94 ± 0.24	4.90 ± 0.36	4.81 ± 0.68	1.06	3.53 ± 1.00	3.93 ± 0.72	−2.86**
Vegetables	4.97 ± 0.21	4.88 ± 0.45	2.15*	4.91 ± 0.35	5.00 ± 0.00	4.98 ± 0.15	2.27	3.63 ± 0.86	3.81 ± 0.80	3.78 ± 1.17	0.54	4.94 ± 0.33	4.86 ± 0.39	1.47
Fish	4.39 ± 0.73	4.27 ± 0.91	1.24	4.15 ± 0.89	4.38 ± 0.61	4.65 ± 0.61	5.50**	2.06 ± 0.78	1.81 ± 0.78	1.95 ± 0.97	1.19	4.32 ± 0.86	4.38 ± 0.64	−0.53
Seafood	3.60 ± 1.01	3.52 ± 1.01	0.66	3.61 ± 1.00	3.51 ± 0.99	3.72 ± 1.05	0.56	4.33 ± 0.83	4.20 ± 0.87	4.29 ± 1.06	0.27	3.56 ± 1.05	3.55 ± 0.82	0.09
Processed meat	1.52 ± 0.78	1.93 ± 0.84	−4.41***	1.94 ± 0.85	1.20 ± 0.44	1.54 ± 0.88	13.62***	4.92 ± 0.28	4.92 ± 0.34	4.78 ± 0.69	1.38	1.70 ± 0.84	1.85 ± 0.81	−1.21
Dairy products	4.02 ± 0.89	3.75 ± 0.93	2.61*	3.74 ± 0.95	3.90 ± 0.91	4.49 ± 0.59	9.74***	3.71 ± 0.96	3.64 ± 0.92	3.49 ± 1.08	0.62	3.86 ± 0.95	3.97 ± 0.77	−0.76
Fruits	4.99 ± 0.12	4.89 ± 0.44	2.70**	4.98 ± 0.15	5.00 ± 0.00	4.98 ± 0.15	0.69	3.27 ± 1.00	2.73 ± 0.91	2.71 ± 1.21	4.65*	4.95 ± 0.32	4.86 ± 0.35	1.94
Cereals	4.47 ± 0.81	4.22 ± 0.91	2.54*	4.30 ± 0.94	4.59 ± 0.67	4.49 ± 0.83	1.66	3.63 ± 0.93	3.39 ± 0.98	3.59 ± 1.14	0.88	4.39 ± 0.84	4.19 ± 0.96	1.55
Legumes	4.87 ± 0.35	4.71 ± 0.60	2.86**	4.85 ± 0.42	4.87 ± 0.34	4.91 ± 0.29	0.32	4.69 ± 0.51	4.80 ± 0.45	4.61 ± 0.83	1.22	4.84 ± 0.46	4.60 ± 0.59	3.29**
Nuts	4.71 ± 0.63	4.43 ± 0.82	3.35***	4.54 ± 0.78	4.72 ± 0.64	4.88 ± 0.32	3.38*	2.37 ± 1.17	3.22 ± 1.15	3.27 ± 1.14	9.47***	4.61 ± 0.73	4.40 ± 0.77	2.01*
Olive oil	4.73 ± 0.64	4.32 ± 0.90	4.46***	4.33 ± 0.90	4.85 ± 0.48	4.98 ± 0.15	15.98***	1.71 ± 0.96	2.20 ± 1.00	2.10 ± 1.04	3.42*	4.57 ± 0.79	4.33 ± 0.85	2.09*
Eggs	4.41 ± 0.76	4.14 ± 0.99	2.67**	4.26 ± 0.93	4.36 ± 0.71	4.65 ± 0.57	3.26*	2.08 ± 1.15	2.70 ± 1.12	2.29 ± 1.06	4.25*	4.29 ± 0.91	4.24 ± 0.80	0.35
Sweets	1.43 ± 0.69	1.46 ± 0.85	−0.33	1.70 ± 0.81	1.25 ± 0.47	1.42 ± 0.73	5.97**	1.12 ± 0.60	1.09 ± 0.28	1.32 ± 0.93	1.82	1.45 ± 0.78	1.45 ± 0.73	0.01
Highly-processed foods	1.05 ± 0.29	1.19 ± 0.68	−2.33*	1.11 ± 0.48	1.00 ± 0.00	1.05 ± 0.21	1.84	1.53 ± 0.94	1.36 ± 0.74	1.54 ± 0.90	0.77	1.12 ± 0.54	1.12 ± 0.46	−0.06
Sugary drinks	1.03 ± 0.16	1.16 ± 0.63	−2.54*	1.02 ± 0.15	1.00 ± 0.00	1.07 ± 0.26	2.43	4.02 ± 0.83	4.22 ± 1.02	4.17 ± 1.12	0.57	1.07 ± 0.40	1.21 ± 0.64	−2.10*
Wine	2.06 ± 1.01	2.38 ± 1.14	−2.60*	1.87 ± 0.96	2.13 ± 1.02	2.16 ± 1.02	1.21	3.86 ± 1.04	4.64 ± 0.64	4.42 ± 0.84	12.04***	2.15 ± 1.06	2.50 ± 1.14	−2.20*
Beer	1.71 ± 0.82	2.01 ± 1.01	−2.89**	1.65 ± 0.85	1.84 ± 0.84	1.58 ± 0.73	1.39	4.18 ± 0.81	4.63 ± 0.72	4.44 ± 0.92	4.04*	1.81 ± 0.90	2.07 ± 1.04	−1.92
Fermented foods (non-alcoholic)	3.16 ± 1.36	2.95 ± 1.22	1.39	2.80 ± 1.33	3.41 ± 1.31	3.19 ± 1.42	2.66	1.16 ± 0.66	1.14 ± 0.57	1.29 ± 0.84	0.69	3.04 ± 1.29	3.14 ± 1.32	−0.53

* p < .05; ** p < .01; *** p < .001.

Fruits, vegetables, legumes, and nuts were consistently rated as the healthiest food groups by both degrees. Although scores were high in all cases, HND students rated these foods significantly higher than FST students, particularly for legumes, nuts, and vegetables, where the mean differences, though small, were statistically significant. Olive oil also stood out as one of the most positively perceived foods across all students, yet HND students again gave it significantly higher scores, confirming its strong association with healthiness among nutrition students (Table 1). At the other end of the scale, processed foods and sugary drinks were the lowest-rated categories. Table 1 shows that even though FST students assigned slightly higher scores, the difference was significant, with both groups clearly identifying these foods as unhealthy and mean ratings close to the lowest value on the scale.

Dairy products, cereals, fish, and eggs were generally perceived as healthy foods, with mean scores above 3.5 in both degrees. HND students tended to give slightly higher ratings for these items, though the differences were not statistically significant (Table 1). Greater variation emerged among animal-based foods. Red meat and processed meat were rated significantly less healthy by HND students compared to FST students, showing one of the largest contrasts between degrees. In contrast, white meat and seafood were evaluated positively by both groups, with moderate to high mean values and no significant differences (see Table 1).

Finally, Table 1 shows that wine and beer received low healthiness ratings from all students, indicating that alcoholic beverages were not perceived as healthy overall. Nonetheless, FST students consistently attributed significantly higher scores to both items than HND students, suggesting a more permissive perception toward alcohol consumption among food science students.

Perceptions of food healthiness evolved notably as students advanced through their degrees (Table 1). Among HND students, statistically significant year-to-year differences were observed in nearly half of the food categories, generally reflecting a progressive refinement of their evaluations over time. Fourth-year students rated healthy foods more positively and unhealthy foods more critically than their first-year peers. In particular, fish, olive oil, nuts, eggs, dairy products, and legumes showed significant increases in perceived healthiness, suggesting a growing awareness of their nutritional value as students progressed through the programme. Conversely, red meat, processed meat, and sweets displayed significant declines in healthiness perception, indicating a clearer alignment with dietary guidance discouraging excessive intake of these foods. Overall, these findings point to a maturation in students’ nutritional judgment and evidence-based reasoning across academic years.

Among FST students, significant differences across years were also identified, though less extensive than among HND students. The perceived healthiness of cereals, nuts, olive oil, fermented foods, wine, and beer increased significantly from the first to the second/third year, before stabilising or slightly declining by the final year. In contrast, red meat was rated significantly less healthy by more advanced students, mirroring the trajectory observed among HND students (see Table 1)

Gender-based comparisons revealed several statistically significant contrasts in how male and female students perceived the healthiness of specific foods (Table 1). Overall, female students rated plant-based and minimally processed foods significantly higher, while male students gave higher ratings to animal-based and alcoholic products.

Table 1 shows that among animal protein sources, red meat and white meat showed the strongest gender differences, with men consistently perceiving them as healthier than women. Conversely, female students assigned significantly higher scores to legumes, nuts, and olive oil, showing stronger adherence to food patterns consistent with public health recommendations.

Finally, wine was the only alcoholic beverage with a significant gender difference, with men rating it slightly healthier than women. Nonetheless, both genders continued to classify alcoholic beverages among the least healthy food categories, reinforcing a broad consensus on their limited contribution to a healthy diet (Table 1).

### Importance of different aspects to consider a food product as healthy

Students were asked to rate the importance of 25 different aspects for a food to be considered healthy, using a Likert scale ranging from 1 (not important) to 5 (very important). In line with the qualitative results, the proposed aspects reflected not only the nutritional dimension but also factors related to production methods, origin, and other attributes influencing perceptions of healthiness.

Across the entire sample, the aspects most strongly associated with a “healthy” food were those related to safety, freshness, and nutritional quality. The highest mean scores corresponded to “compliance with sanitary controls,” “freshness,” and “rich in vitamins, minerals, and essential nutrients,” all scoring above 4 in both degrees. This indicates a clear consensus that food safety and nutrient adequacy are central criteria for defining food healthiness (Table 2).

**Table 2:** Importance given to different aspects to consider a food product as healthy by bachelor’s degree, year and gender.

Aspect	HND (n = 151)	FST (n = 149)		HND				FST				Female (n = 241)	Male (n = 58)	
	(M ± SD)	(M ± SD)	t	1^st^ (n = 46) (M ± SD)	2^nd^/3^rd^ (n = 61) (M ± SD)	4^th^ (n = 44) (M ± SD)	F	1^st^ (n = 49) (M ± SD)	2^nd^/3^rd^ (n = 59) (M ± SD)	4^th^ (n = 41) (M ± SD)	F	(M ± SD)	(M ± SD)	t
Low in fat	2.79 ± 1.10	3.22 ± 0.99	−3.59***	2.85 ± 1.01	2.92 ± 1.20	2.53 ± 1.01	1.66	3.35 ± 0.90	2.98 ± 1.03	3.42 ± 1.00	2,95	2.98 ± 1.10	3.12 ± 0.92	−0.93
Sugar-free	3.77 ± 1.16	3.67 ± 1.00	0.77	3.54 ± 1.21	3.90 ± 1.17	3.81 ± 1.08	1.32	3.61 ± 1.00	3.68 ± 0.92	3.73 ± 1.12	0,16	3.74 ± 1.09	3.62 ± 1.02	0.77
Gluten-free	1.53 ± 0.86	1.75 ± 1.08	−1.94	1.80 ± 0.98	1.56 ± 0.89	1.19 ± 0.50	6.27**	1.82 ± 1.19	1.86 ± 1.03	1.49 ± 1.00	1,65	1.62 ± 0.98	1.69 ± 0.98	−0.47
Lactose-free	1.76 ± 1.17	1.87 ± 1.15	−0.84	2.13 ± 1.29	1.74 ± 1.17	1.40 ± 0.90	4.63*	1.96 ± 1.26	2.00 ± 1.08	1.59 ± 1.10	1,79	1.85 ± 1.19	1.69 ± 1.01	0.92
Low in calories	2.22 ± 1.08	2.83 ± 1.09	−4.83***	2.57 ± 0.96	2.28 ± 1.23	1.77 ± 0.81	6.69**	2.96 ± 1.14	2.66 ± 1.03	2.90 ± 1.11	1,15	2.46 ± 1.12	2.78 ± 1.11	−1.93
Wholegrain	4.20 ± 0.89	3.50 ± 1.11	6.06***	3.70 ± 1.13	4.51 ± 0.60	4.30 ± 0.71	13.18***	3.29 ± 1.06	3.66 ± 1.12	3.51 ± 1.12	1,56	3.98 ± 1.00	3.29 ± 1.14	4.59***
Plant-based	3.59 ± 1.28	3.22 ± 1.29	2.55*	3.52 ± 1.46	3.61 ± 1.27	3.65 ± 1.11	0.12	3.18 ± 1.25	3.19 ± 1.28	3.29 ± 1.38	0,10	3.61 ± 1.22	2.57 ± 1.29	5.75***
Animal welfare	3.92 ± 1.16	3.80 ± 1.23	0.88	4.20 ± 1.03	3.71 ± 1.27	3.93 ± 1.08	2.41	3.61 ± 1.35	4.02 ± 1.08	3.71 ± 1.25	1,63	4.00 ± 1.12	3.29 ± 1.30	4.15***
Natural	4.09 ± 0.96	4.08 ± 1.06	0.05	4.22 ± 0.81	4.12 ± 1.02	3.91 ± 1.02	1.20	4.16 ± 0.97	4.09 ± 0.93	3.98 ± 1.33	0,35	4.19 ± 0.93	3.66 ± 1.22	3.67***
Fresh	4.38 ± 0.77	4.34 ± 0.84	0.41	4.28 ± 0.75	4.49 ± 0.70	4.33 ± 0.89	1.11	4.45 ± 0.58	4.37 ± 0.74	4.17 ± 1.16	1,31	4.42 ± 0.76	4.12 ± 0.96	2.56*
Industrial	2.77 ± 1.30	2.79 ± 1.24	−0.08	2.89 ± 1.23	2.53 ± 1.29	3.00 ± 1.35	2.00	3.12 ± 1.27	2.80 ± 1.19	2.37 ± 1.20	4,32	2.81 ± 1.30	2.66 ± 1.15	0.83
Local / Km0	3.39 ± 1.12	3.41 ± 1.22	−0.12	3.37 ± 1.20	3.31 ± 1.09	3.54 ± 1.10	0.51	3.25 ± 1.22	3.56 ± 1.15	3.39 ± 1.32	0,90	3.48 ± 1.16	3.09 ± 1.19	2.30*
Rich in vitamins, minerals, and essential nutrients	4.29 ± 0.83	4.18 ± 0.87	1.14	4.28 ± 0.86	4.41 ± 0.67	4.14 ± 0.99	1.34	4.06 ± 0.94	4.36 ± 0.74	4.07 ± 0.93	2,00	4.23 ± 0.88	4.26 ± 0.74	−0.21
Seasonal	4.13 ± 0.99	3.95 ± 1.07	1.57	3.89 ± 1.02	4.26 ± 1.02	4.21 ± 0.89	2.06	3.82 ± 1.05	4.05 ± 1.01	3.95 ± 1.18	0,64	4.16 ± 0.94	3.53 ± 1.23	4.27***
Organic	3.12 ± 1.11	3.32 ± 1.16	−1.49	3.48 ± 1.15	3.12 ± 1.05	2.74 ± 1.03	5.18**	3.63 ± 1.01	3.29 ± 1.16	2.98 ± 1.26	3,72*	3.28 ± 1.13	2.95 ± 1.12	2.02*
Home-cooked	4.06 ± 0.98	3.97 ± 1.03	0.75	3.85 ± 1.05	4.20 ± 0.91	4.09 ± 0.97	1.72	4.27 ± 0.73	4.09 ± 0.90	3.46 ± 1.33	7,99***	4.08 ± 0.97	3.78 ± 1.13	2.04*
Without preservatives or sweeteners	3.46 ± 1.11	3.08 ± 1.25	2.78**	3.67 ± 0.99	3.33 ± 1.14	3.42 ± 1.18	1.32	3.63 ± 1.17	2.92 ± 1.01	2.66 ± 1.44	8,40***	3.33 ± 1.22	3.03 ± 1.08	1.68
Compliance with sanitary controls	4.51 ± 0.81	4.75 ± 0.63	−2.85**	4.63 ± 0.65	4.53 ± 0.79	4.37 ± 0.98	1.15	4.71 ± 0.65	4.85 ± 0.41	4.66 ± 0.83	1,24	4.63 ± 0.73	4.62 ± 0.75	0.13
Prevent chronic diseases	3.84 ± 1.12	3.77 ± 1.12	0.53	3.83 ± 1.04	4.03 ± 1.06	3.58 ± 1.26	2.07	4.14 ± 0.87	3.88 ± 1.08	3.17 ± 1.20	10,04***	3.77 ± 1.14	3.97 ± 1.04	−1.21
Do not cause weight gain	2.35 ± 1.18	2.69 ± 1.17	−2.44*	2.46 ± 1.22	2.41 ± 1.22	2.16 ± 1.07	0.81	3.00 ± 1.08	2.41 ± 1.13	2.71 ± 1.25	3,58*	2.52 ± 1.21	2.53 ± 1.06	−0.12
Pleasant / Tasty	4.01 ± 1.11	3.99 ± 1.11	0.10	4.17 ± 0.95	4.08 ± 1.12	3.72 ± 1.24	2.10	4.18 ± 0.99	4.05 ± 1.04	3.68 ± 1.27	2,47	4.04 ± 1.10	3.83 ± 1.14	1.32
Traditional	3.03 ± 1.08	3.05 ± 1.13	−0.16	2.98 ± 1.13	3.26 ± 1.12	2.77 ± 0.92	2.78	3.04 ± 1.14	3.12 ± 1.15	2.98 ± 1.11	0,20	3.06 ± 1.10	2.98 ± 1.12	0.47
Known origin	3.67 ± 1.09	3.76 ± 1.16	−0.65	3.72 ± 1.09	3.72 ± 1.11	3.56 ± 1.08	0.33	3.67 ± 1.13	3.81 ± 1.12	3.78 ± 1.28	0,20	3.77 ± 1.06	3.48 ± 1.35	1.76
Self-produced	3.22 ± 1.25	3.34 ± 1.24	−0.80	3.20 ± 1.36	3.38 ± 1.27	3.02 ± 1.08	1.03	3.49 ± 1.08	3.49 ± 1.32	2.93 ± 1.23	3,17*	3.38 ± 1.20	2.86 ± 1.34	2.87**
GMO-free	2.75 ± 1.17	2.75 ± 1.19	−0.04	2.98 ± 1.18	3.02 ± 1.18	2.12 ± 0.91	9.78***	3.00 ± 1.17	2.92 ± 1.13	2.22 ± 1.13	6,18**	2.75 ± 1.15	2.76 ± 1.28	−0.07

* p < .05; ** p < .01; *** p < .001.

In contrast, exclusionary or marketing-driven claims, such as gluten-free, lactose-free, or prevents weight gain, were considered the least relevant, with mean ratings around 1.5 to 2.5. These results, shown in Table 2, suggest that students prioritised the positive attributes of foods (nutritional value, naturalness, safety) over restrictive claims when assessing healthiness.

When comparing degrees, statistically significant differences reflected the disciplinary focus of each programme. FST students assigned greater importance to aspects linked to energy and weight management, particularly low in fat, low in calories, and prevents weight gain, all showing significant differences in favour of this group. In contrast, HND students rated compositional and production-related characteristics significantly higher, including wholegrain, plant-based, and absence of preservatives or sweeteners. They also valued compliance with sanitary controls more strongly, showing a clearer emphasis on quality and formulation aspects grounded in public-health nutrition (see Table 2).

Among HND students, statistically significant year effects were identified for six aspects: gluten-free, lactose-free, low in calories, wholegrain, organic, and GMO-free. First-year students rated gluten-free, lactose-free, and low in calories significantly higher than fourth-year students, indicating a gradual decline in the perceived importance of these exclusionary or label-related attributes across academic progression. In contrast, the importance assigned to wholegrain increased markedly throughout the degree, with fourth-year students giving significantly higher ratings than both first- and second/third-year students (Table 2).

Table 2 also shows that for organic and GMO-free, first-year students also gave higher scores than fourth-year students, though mean values remained moderate overall. 

Among FST students, statistically significant differences across academic years were found for eight aspects: GMO-free, organic, industrial, home-cooked, without preservatives or sweeteners, chronic-disease prevention, prevents weight gain, and low in calories (see Table 2).

First-year students assigned significantly higher importance to GMO-free and organic compared with fourth-year students, while the importance of industrial decreased progressively over the years, showing the lowest mean ratings in the final year. For home-cooked and without preservatives or sweeteners, first-year and second/third-year students scored significantly higher than fourth-year students (Table 2). Similarly, the aspects of chronic-disease prevention, prevents weight gain, and low in calories declined significantly between the first and later years, with fourth-year students giving consistently lower ratings for these aspects (Table 2).

Gender-based comparisons revealed statistically significant differences for ten of the evaluated aspects (Table 2). Although both male and female students agreed on the central role of safety, freshness, and nutrient content, female students consistently rated significantly higher a wide range of attributes related to naturalness, sustainability, and ethical production. This included significantly higher importance given to “plant-based origin,” “animal welfare,” “wholegrain,” “natural,” “fresh,” “local/Km0,” “seasonal,” “organic,” “home-cooked,” and “self-produced” products. Male students did not rate any aspect significantly higher than female students.

### Aspects that increase trust when purchasing food products

**Fig 2:**
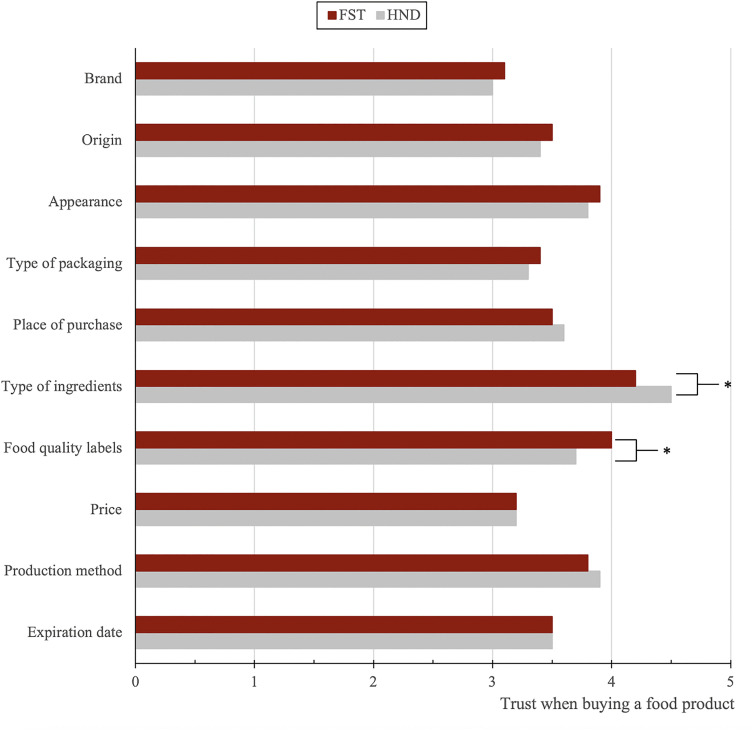
Comparison of mean Likert scores for factors influencing trust when purchasing a food product between HND and FST students. * p < .05.

Students were asked to rate how much trust or confidence they placed in different aspects when buying a food product. Overall, the most trusted aspects among both bachelors were the “Type of ingredients used” (M = 4.49 HND, 4.23 FST), the “Way of production” (M = 3.81 HND, 3.74 FST), and the “Presence of official quality seals” (M = 3.67 HND, 4.05 FST). Conversely, “Brand” (M = 2.97 HND, 3.11 FST), and “Price” (M = 3.22 HND, 3.21 FST), were the lowest-rated aspects in terms of building trust in food products (Fig 2).

Statistical analysis confirmed two significant differences (Fig 2). First, HND students attributed significantly more trust to the type of ingredients (t = 3.086, p = .002, d = 0.36), compared to FST students, reflecting a small-to-moderate effect size and indicating a meaningful but not large difference between degrees. Second, students from FST trusted quality seals significantly more than HND students (t = −3.227, p = .001, d = 0.37), again representing a small-to-moderate effect.

When analysing the results of this aspect by year of the bachelor’s degree, statistically significant differences were found in only one item for each programme. Among HND students, significant differences were only observed for “Expiration date” (F = 4.663, p = .011, η² = 0.06). First-year students (M = 3.96, SD = 0.87) rated this aspect as more important than both second/third-year students (M = 3.44, SD = 1.04) and fourth-year students (M = 3.33, SD = 1.25) (Fig 2), with the effect size indicating a small-to-moderate year-related difference and a gradual decline in importance across academic progression.

Fig 2 shows that for FST students, “Method of production” was rated differently across years (F = 4.251, p = .016, η² = 0.06), with first-year (M = 3.86, SD = 0.74) and second/third-year students (M = 3.88, SD = 0.97) assigning greater importance than fourth-year students (M = 3.39, SD = 0.97). This small-to-moderate effect suggests a consistent reduction in the relevance attributed to production methods in later stages of the degree.

The comparison of scores between male and female students regarding the aspects that inspire trust or confidence when buying a food product revealed statistically significant gender differences for two of the included items. For the “Type of packaging”, female students assigned significantly higher importance (M = 3.50, SD = 0.94) compared to male students (M = 3.19, SD = 0.89) (t = 2.296, p = .022, d = 0.34), reflecting a small-to-moderate effect. A statistically significant difference was also observed for “Food quality seals”, where male students considered them more important (M = 4.19, SD = 0.81) than female students (M = 3.78, SD = 1.05) (t = −2.785, p = .006, d = 0.41), again indicating a small-to-moderate gender difference (see Fig 2).

## Discussion

This study explored undergraduate conceptions of food healthiness across HND and FST bachelor degrees, integrating qualitative narratives and quantitative evaluations. Students largely converged around moderation-based, nutrition-focused conceptualizations. However, subgroup differences offer actionable insights for curriculum design and professional training. These patterns echo earlier degree-specific findings, while providing finer resolution on gradients relevant to educational planning and future practice [24,27,29].

Students expressed strong agreement with both biomedical and holistic health-oriented definitions of food, underscoring the centrality of physiological functions and overall well-being in their conceptualisations. The view of food as a source of energy, nutrients, and a driver of physical, mental, and emotional health was the most endorsed statement across both HND and FST students, confirming that biomedical rationales remain foundational in how they frame the meaning of food. Such emphasis also resonates with the concept of nutritionism, as described by Scrinis (2013) [13], whereby food is primarily valued for its nutrient composition and physiological effects, often overshadowing symbolic, cultural, or experiential dimensions.

By contrast, the lower and progressively weakening endorsement of symbolic, cultural, and communicative definitions of food suggests that university training may contribute to a reorientation of students’ conceptual frameworks toward more technical and medicalized modes of understanding. Rather than simply reflecting differences between groups, this pattern points to a possible process of professional socialisation in which medicalized and functional criteria become increasingly dominant, while broader sociocultural and integrative perspectives lose salience [30]. Such attenuation of sociocultural and holistic views might be concerning, given that these dimensions have been recognised as critical for understanding food practices and promoting healthy and sustainable dietary transitions [27,31,32]. If educational trajectories in food-related disciplines progressively marginalise these perspectives, there is a risk of preparing professionals who are highly skilled in biomedical and technological domains but less equipped to integrate social, cultural, and ethical dimensions into dietary guidance and food system innovation.

Gender differences were equally salient in the conceptualization of food. Women expressed higher agreement with both sociocultural definitions and holistic framings that incorporate physical, mental, and emotional health, while men leaned more toward the biomedical definition. This also aligns with prior work showing that women are more likely to acknowledge symbolic and relational aspects of eating, incorporating food’s role in identity, care, and social connection, whereas men are more inclined to view food through utilitarian and physiological lenses [33,34].

Overall, the qualitative and quantitative findings illustrate that the conceptions of food healthiness of students are shaped not only by scientific knowledge but also by cultural discourses, gendered values, and professional socialisation. Focus-group narratives indicate that students draw on a dominant “moderation and balance” repertoire when defining healthy eating, which reflects a biomedical framing of food [35] and aligns with nutritionism’s tendency to privilege nutrients and physiological functions over pleasure, commensality, and cultural meaning [13]. Importantly, the recurrent claim in focus group discussions that “it depends on the person” suggests an individualised understanding of healthiness that may coexist with categorical moral evaluations of foods, highlighting a tension between relativism in principle and hierarchy in practice. This tension is central to social representations of food healthiness and helps explain why students can endorse nuanced definitions while still reproducing stable “healthy/unhealthy” rankings [36].

To contextualise these results, it is also instructive to compare them with evidence from Spain. A qualitative study of adolescents in Spain explored how young people conceptualise healthy eating, and found that although relationally the adolescents recognized nutritional elements (balance, diversity, limiting excess fats or sugars), social, cultural, emotional, and environmental factors significantly influenced definitions and practices [37]. Similarly, in a Spanish university community, adherence to the Mediterranean Diet was moderate overall, with nearly 41% of participants showing low adherence, and practical and cultural constraints limiting alignment with ideal behaviours [35]. These findings mirror the present results in several respects. First, they suggest that although participants were aware of general notions of healthy eating (such as balance, variety, and moderation) their actual conceptions and practices [24] were also mediated by contextual, social, and emotional factors rather than by technical or scientific knowledge. Second, both studies highlight how perceptions of healthy eating differ across social and demographic subgroups, reflecting the influence of sociocultural environments and life stages.

With regard to the analysis of different foods as healthy, a clear consensus can be observed among students from both degrees, with strong valorisation of olive oil, vegetables, legumes, fruits and nuts, in contrast to sweets, sugary drinks, and highly processed foods. Where groups diverged, differences were most visible for animal-derived foods. These findings are consistent with previous research in student populations, where plant-based staples are consistently positioned at the top of health hierarchies and sugary or highly-processed items at the bottom [27].

In addition, the conceptions of healthy eating or healthy food are produced and shaped according to cultural contexts [6]. The prominence of foods such as olive oil, legumes, and nuts in students’ healthiness hierarchies should be interpreted in light of the Mediterranean food cultures in which the study is embedded. These foods are not only supported by nutritional evidence but are also culturally valorised and symbolically associated with health within Southern European dietary traditions [6,8,10]. Cross-cultural studies on food perceptions among health professionals from different countries have shown that, even though they have scientific knowledge about food, their perceptions and recommendations are influenced by their cultural norms and values, and that there is a dialectical relationship between nutritional norms and sociocultural norms [6,38]. Therefore, cross-cultural comparative studies would be needed to further examine how professional training interacts with local food cultures in shaping social representations of healthy eating.

The shared hierarchy of foods in both degrees suggests that students enter university with widely circulating public-health and media scripts about “healthy food,” which are then refined rather than fundamentally reconfigured through training. The year-of-study gradients suggest that training consolidates particular evidence-based constructs, such as increased scepticism toward processed meats and greater endorsement of foods aligned with dietary guidance.

Gender differences regarding the analysis of different foods as healthy were consistent with documented sociocultural gendered food norms. Rather than reflecting only knowledge differences, the findings mirror well-established sociocultural patterns in which women’s diets are more closely aligned with health-oriented and plant-based consumption, whereas men continue to valorize meat and alcohol [33,39]. Sociological studies have long linked meat consumption with hegemonic masculinity and the reinforcement of traditional gender roles [34,40–43]. Conversely, women have traditionally been expected to favour “lighter,” “delicate,” or “clean” foods, such as white meats or plant-based options, which are culturally connected to femininity, restraint, and body care. Women are still socially positioned as responsible for family feeding practices and household nutrition, which may enhance their nutritional knowledge and increase the salience of health responsibility in their dietary decisions [43]. This aligns with women’s stronger endorsement of plant-based and “nutritionally dense” foods as well as greater concern for weight control [33,44,45]. Such normative pressures may explain why female students consistently gave higher healthiness ratings to foods symbolically associated with healthiness, “lightness” and well-being.

Recent survey-based evidence across European contexts also confirms these trends. Feraco et al. (2024) reported that, in a large Italian cohort, men preferred red and processed meat and alcohol, while women showed greater preference for vegetables, whole grains, soy products, and high-cocoa dark chocolate, aligning with healthier dietary patterns [34]. Comparable gender differences were also identified by Ruby et al. (2016), who found that across Argentina, Brazil, France, and the United States, men expressed more positive attitudes toward beef, reported greater liking and craving, and consumed beef more frequently than women [40]. The gender differences in perceptions of alcoholic beverages (wine and beer) observed in the present study also reflect similar findings in population-level surveys, where men report greater alcohol consumption and perceive moderate intake as compatible with a healthy lifestyle [33,41]. In this sense, food healthiness operates as a cultural and identity-laden judgement, meaning that educational interventions focused solely on nutritional knowledge may be insufficient unless they also address the gendered meanings attached to specific foods and practices.

The attributes associated by students to define a “healthy food” were grounded mainly in ideas of safety, freshness, and nutritional value, while exclusionary claims played a minor role and trust was placed primarily in ingredients, production methods, and quality seals.

The contrast between HND and FST students reflects their disciplinary lenses: FST students tended to frame healthiness through energy-related attributes and institutional guarantees, whereas HND students emphasised compositional and production characteristics and relied more on the nature of ingredients. These differences offer a useful basis to explore how each group interprets the role of processing, safety governance, and industrial practices when constructing their understanding of what makes a food “healthy.” Differences in how processing is interpreted are particularly relevant for professional practice. When processing is primarily associated with chemicals and distrust, risk communication may become vulnerable to simplistic narratives that always equate industrial production with harm [46]. Conversely, FST students’ stronger reliance on regulation, standards, and official seals suggests a more institutionalised trust model. Curricula may therefore benefit from explicitly teaching how safety, processing, and additives are governed, while also addressing how lay concerns emerge and how professionals can better communicate to the population.

Clear gender-based differences also emerged in the perception of students’ attributes of healthy products and sources of trust. Female students consistently emphasized naturalness, freshness, seasonality, proximity, and organic production, while also assigning higher importance to animal welfare and plant-based criteria. This pattern echoes evidence that women more frequently integrate ethical and sustainability-oriented values into food choice, reflecting heightened sensitivity to ecological and moral dimensions of eating [14,27,33]. By contrast, men tended to attach greater value to institutional cues, such as official quality seals, suggesting a stronger reliance on external authorities rather than everyday experiential indicators when constructing trust. These findings resonate with risk perception studies showing that women display greater concern for food safety, transparency, and environmental risks, whereas men report higher trust in regulatory systems and industrial standards [12,41].

The intersection of gender with sustainability transitions warrants particular attention. The stronger valuation of plant-based diets, animal welfare, and ecological attributes from female students aligns closely with current health and sustainability recommendations [18,19], suggesting that women may exhibit greater willingness and preparedness to implement dietary changes aligned with sustainability goals. As highlighted in previous studies, these differences may stem from historically constructed gender roles that shape food-related practices and meanings [30]. Women have traditionally been responsible for the family care and the acquiring and preparing food, which has provided them with greater practical experience and a deeper understanding of healthy eating. Empathy and caring, especially through food, are values culturally associated with femininity [44,45], and women tend to seek more information about food and nutrition, engage in dieting more often, and display higher sensitivity toward environmental, animal welfare, and health aspects of food [30,34,47]. Recognizing and addressing gender asymmetries is crucial for designing interventions that not only challenge gender stereotypes but also harness gender-specific motivations to accelerate transitions toward healthier and more sustainable diets and fairer societies.

From an educational perspective, the findings of the present study suggest the need to more explicitly integrate and valorise knowledge from the social sciences, such as anthropology and sociology of food, more explicitly and systematically within undergraduate nutrition and food science curricula. Issues related to food, the body, and health, including the very construction of what is considered “healthy eating,” are not solely biological but are also shaped by sociocultural, economic, ethical, and political factors [10,39,48]. As argued by Lordly et al. (2019) in the context of nutrition, a “critical dietetic education” is needed to move beyond the dominant positivist paradigm and to integrate insights from the humanities and social sciences, enabling a more multidimensional and transtheoretical understanding of food and health [49]. Embedding sociocultural, critical nutrition, and sustainability-oriented perspectives from the earliest years of training, together with practical learning activities that expose students to diverse social and cultural realities, could help counterbalance the progressive dominance of purely biomedical or technological framings while fostering greater sociocultural awareness and critical perspective. In this context, the disciplinary contrasts observed between HND and FST students further support the value of interdisciplinary teaching activities, fostering dialogue between future nutritionists and food technologists and promoting more integrated professional perspectives on healthy and sustainable food systems.

This research presents certain methodological limitations that should be taken into account when interpreting the findings. First, the single-institution design limits generalisability and makes it difficult to disentangle patterns related to the specific curricular and pedagogical orientations of this university from those reflecting broader sociocultural or disciplinary trends. Multi-centre studies including universities from different regions and countries would be needed to assess the transferability of the observed representations. Second, the high proportion of female participants, although typical of nutrition and health-related degrees [50,51], may have influenced the overall patterns and the magnitude of gender differences, potentially amplifying perspectives more prevalent among women and limiting the representativeness of male viewpoints. Finally, voluntary participation may have introduced self-selection and social desirability biases. Students more interested in health or sustainability may have been over-represented, and some participants may have reproduced “textbook” or guideline-consistent definitions of healthy eating to appear professionally competent, particularly in the focus group setting. Consequently, their answers may partly reflect normative or performative discourses rather than the full diversity of students’ lived conceptualisations. Future research using multi-institutional designs, more gender-balanced samples, random or classroom-based recruitment, and complementary qualitative approaches would help to mitigate these biases and capture a wider range of everyday representations.

## Conclusions

This study provides novel insights into how undergraduate students in NHD and FST conceptualise the healthiness of foods. Findings demonstrate that students predominantly adopt a moderation- and nutrient-based view, but this is tempered by categorical judgements, production-related cues, and gendered values.

Training exerts a cumulative effect, consolidating evidence-based constructs, while attenuating reliance on simplified production-related cues. At the same time, endorsement of sociocultural and holistic perspectives declined, particularly among advanced FST students, suggesting that curricula may inadvertently narrow conceptualisations of food toward biomedical framings. Gender differences further underscored how ethical, sustainability-related, and institutional trust cues are unevenly valued.

Together, these findings highlight the need for curricula that integrate biomedical, sociocultural, and sustainability perspectives more consistently across training trajectories. Doing so would better equip future dietitians and food technologists to address the multifaceted nature of food healthiness and to communicate effectively in contexts where scientific, cultural, and ethical discourses intersect.
